# Elevated murine HB-EGF confers sensitivity to diphtheria toxin in EGFR-mutant lung adenocarcinoma

**DOI:** 10.1242/dmm.049072

**Published:** 2021-11-15

**Authors:** Camila Robles-Oteiza, Deborah Ayeni, Stellar Levy, Robert J. Homer, Susan M. Kaech, Katerina Politi

**Affiliations:** 1 Department of Immunobiology, Yale School of Medicine, New Haven, CT 06510, USA; 2 Department of Pathology, Yale School of Medicine, New Haven, CT 06510, USA; 3 Yale Cancer Center, Yale School of Medicine, New Haven, CT 06510, USA; 4 NOMIS Center for Immunobiology and Microbial Pathogenesis, Salk Institute, La Jolla, CA 92037, USA; 5 Department of Medicine (Section of Medical Oncology), Yale School of Medicine, New Haven, CT 06510, USA

**Keywords:** Lung adenocarcinoma, Diphtheria toxin, DTR, HBEGF, EGFR

## Abstract

Conditional ablation of defined cell populations *in vivo* can be achieved using genetically engineered mice in which the human diphtheria toxin (DT) receptor (DTR) is placed under control of a murine tissue-specific promotor, such that delivery of DT selectively ablates cells expressing this high-affinity human DTR; cells expressing only the endogenous low-affinity mouse DTR are assumed to be unaffected. Surprisingly, we found that systemic administration of DT induced rapid regression of murine lung adenocarcinomas that express human mutant *EGFR* in the absence of a transgenic allele containing human DTR. DT enzymatic activity was required for tumor regression, and mutant *EGFR*-expressing tumor cells were the primary target of DT toxicity. In FVB mice, *EGFR*-mutant tumors upregulated expression of HBEGF, which is the DTR in mice and humans. HBEGF blockade with the enzymatically inactive DT mutant CRM197 partially abrogated tumor regression induced by DT. These results suggest that elevated expression of murine HBEGF, i.e. the low-affinity DTR, confers sensitivity to DT in *EGFR*-mutant tumors, demonstrating a biological effect of DT in mice lacking transgenic DTR alleles and highlighting a unique vulnerability of *EGFR*-mutant lung cancers.

## Abstract (precis)

**Editor's choice:** We report the unexpected finding that systemic administration of diphtheria toxin induces rapid regression of murine lung adenocarcinomas expressing human *EGFR^L858R^* in the absence of a transgenic allele containing human diphtheria toxin receptor.

## INTRODUCTION

Conditional ablation of defined cell populations *in vivo* is an essential strategy to probe cell function. In mice, cells can be efficiently ablated using diphtheria toxin (DT), an exotoxin secreted by *Corynebacterium diphtheriae* (Hadfield et al., 2000). This approach relies on human DT receptor (DTR)'s ∼10^5^-fold higher affinity for DT compared to mouse DTR (Mekada et al., 1982; Pappenheimer et al., 1982; Saito et al., 2001). Murine cells that express this high-affinity human DTR under the control of a tissue-specific promoter are sensitive to DT, whereas murine cells that express their endogenous – i.e. low-affinity – DTR are assumed to be unaffected (Saito et al., 2001). In sensitive cells, DT inhibits protein synthesis by inactivating elongation factor 2 (EEF2) and inducing apoptotic cell death (Honjo et al., 1968; Robinson et al., 1974; Morimoto and Bonavida, 1992). In addition to the enzymatic domain that mediates toxicity, DT contains receptor-binding and translocation domains that mediate entry into the cytoplasm of the host cell (Greenfield et al., 1983). The receptor for DT in mice and humans is the membrane-bound precursor of heparin-binding EGF-like growth factor (HBEGF) (Naglich et al., 1992).

DTR-based cell ablation systems are widely used to investigate the roles of defined cell types in disease (Saito et al., 2001; Jung et al., 2002; Kim et al., 2007; Walzer et al., 2007; Whiddon and Palmiter, 2013; Yamazaki et al., 2013). However, upon DT administration, wild-type (WT) mice lacking transgenic DTR alleles exhibit phenotypes including weight loss (Meyer Zu Hörste et al., 2010), proteinuria (Goldwich et al., 2012) and cochlear damage (Konishi et al., 2017). Another study detected lung inflammation after DT administration in both C57BL/6 Foxp3^DTR^ (Kim et al., 2007) and WT animals (Chapman and Georas, 2013). Although these effects are considered ‘off-target’ in studies that rely on DTR transgenic animals, they demonstrate that DT has biological effects in WT mice.

We report that DT induces regression of murine lung adenocarcinomas that express human mutant *EGFR* in the absence of a transgenic DTR allele. By using the inactive DT mutant cross-reacting material 197 (CRM197), we confirmed that enzymatic activity of DT is required for apoptotic tumor cell death. We found that *EGFR*-mutant tumors upregulate expression of the DT receptor, HBEGF, and our data indicate that elevated levels of murine HBEGF confer sensitivity to DT.

## RESULTS

### Murine lung adenocarcinomas expressing human mutant EGFR are sensitive to DT treatment

We initially investigated the role of forkhead box protein P3-positive (Foxp3^+^) regulatory T cells (Tregs) in *EGFR*-mutant lung adenocarcinomas. We used *TetO-EGFR^L858R^; CCSP-rtTA* mice, in which expression of the human *EGFR^L858R^* point mutant can be induced in type II lung epithelial cells by administration of doxycycline (dox) to induce multifocal lung adenocarcinomas (Politi et al., 2006). We crossed these animals with *Foxp3^IRES-DTR-GFP^* mice, in which DT administration depletes Foxp3^+^ Tregs (Kim et al., 2007). Between 6 and 7 weeks after induction with dox, mice received 0.5 µg DT or vehicle control (PBS) by intraperitoneal (i.p.) injection. Magnetic resonance imaging (MRI) revealed that, unlike vehicle controls, DT-treated tumors regressed to ∼30% of the starting volume after 11 days (Fig. S1A). We confirmed that DT depleted Foxp3^+^ Tregs in tumor-bearing lungs (Fig. S1B). Consistent with prior studies (Kim et al., 2007; Joshi et al., 2015), Treg depletion increased CD8^+^ and CD4^+^Foxp3^−^ T cell abundance (Fig. S1C). To verify that Treg depletion was indeed the mechanism by which tumors regressed after DT, we treated tumor-bearing *Foxp3^WT^* mice with DT. Surprisingly, tumors regressed in mice lacking the DTR allele (Fig. S1A). We also found that a single dose of DT was sufficient to induce rapid, yet reversible, tumor regression (Fig. S1D).

**Fig. 1.**
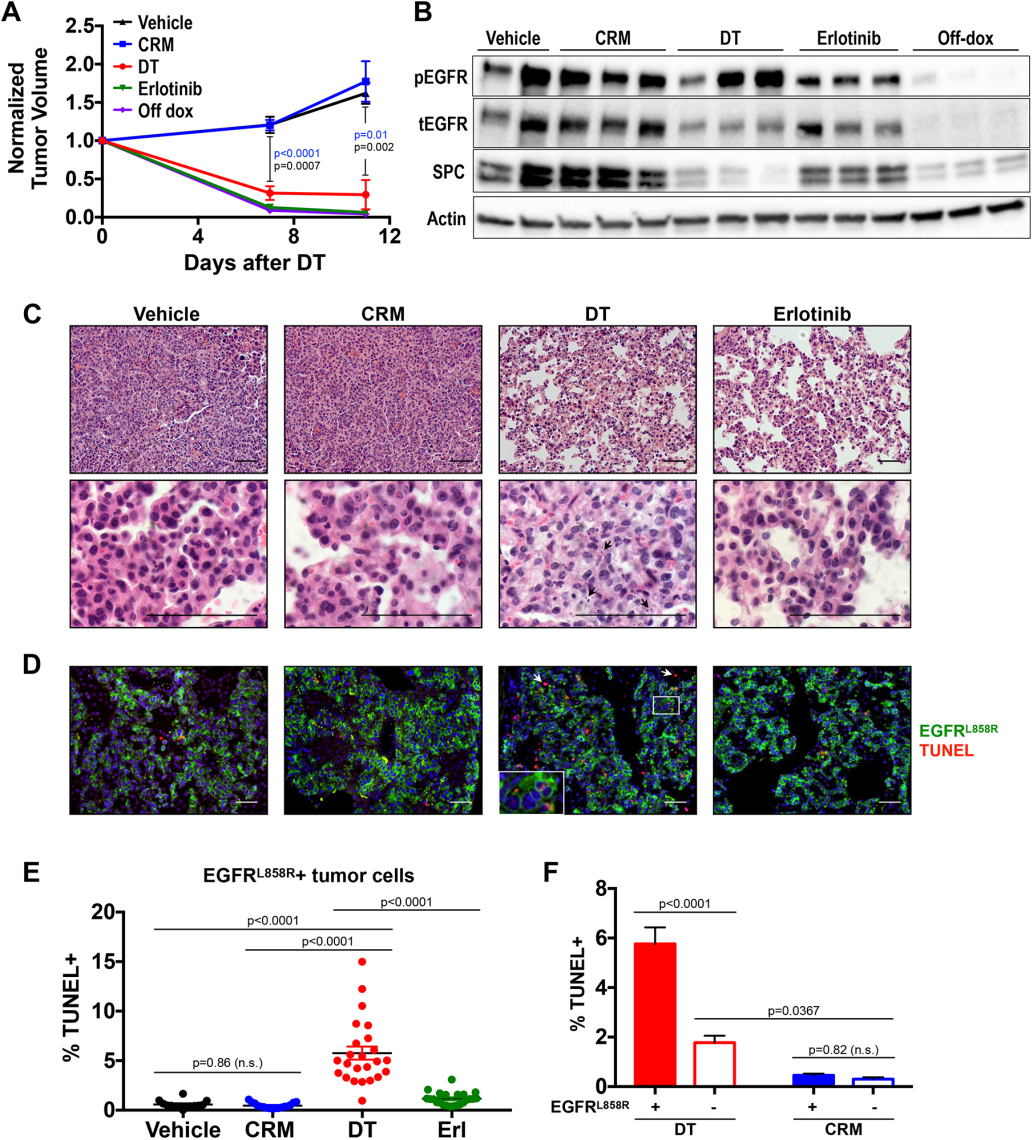
**DT induces regression of murine EGFR^L858R^ lung adenocarcinomas.** (A-C) Tumor-bearing FVB *TetO-EGFR^L858R^; CCSP-rtTA* mice were treated with PBS (vehicle; *n*=5), CRM197 (CRM; *n*=8), diphtheria toxin (DT; *n*=5) or erlotinib (*n*=4), or were taken off doxycycline diet (Off dox; *n*=4), for 11 days. (A) Tumor growth curves quantified from magnetic resonance (MR) images and normalized to tumor volume before treatment; *P*-values indicate treatment with vehicle (black) or CRM197 (blue) compared to treatment with DT. (B) Western blot of lung tumor lysates immunostained for phosphorylated EGFR (pEGFR), total EGFR (tEGFR) and SPC. (C) Representative H&E staining of lung tumor sections; bottom images show remaining tumor cell clusters. (D-F) Tumor-bearing FVB *TetO-EGFR^L858R^; CCSP-rtTA* mice were treated with vehicle control (*n*=2), CRM197 (*n*=3), DT (*n*=3) or erlotinib (*n*=3) for 2 days. (D) Immunofluorescence staining for EGFR^L858R^ and TUNEL in representative lung tumors. Inset shows three TUNEL+ EGFR^L858R^+ (tumor) cells; arrows show TUNEL+ EGFR^L858R^− (non-tumor) cells. (E,F) Percentage of EGFR^L858R^+ (tumor) cells that are TUNEL+ (E) and percentage of EGFR^L858R^+ (tumor) and EGFR^L858R^− (non-tumor) cells that are TUNEL+ (F), quantified using CellProfiler from four to ten fields of view (FOV) per mouse. Scale bars: 50 µm. Results are shown as mean±s.e.m. with *P*-values calculated using unpaired Student's *t*-tests (A) or one-way ANOVA (E,F). n.s., not significant.

To understand how DT could induce tumor regression in mice lacking DTR alleles, we treated tumor-bearing FVB *TetO-EGFR^L858R^; CCSP-rtTA* mice with 0.5 µg DT or CRM197, a DT mutant harboring a single amino acid substitution (G52E) in the catalytic domain. This mutation renders CRM197 inactive, yet it retains an intact receptor-binding domain (Uchida et al., 1973). We compared these agents to the tyrosine kinase inhibitor erlotinib or to dox withdrawal, which both induce rapid tumor regression by inhibiting EGFR phosphorylation and turning off oncogenic *EGFR^L858R^*, respectively (Politi et al., 2006). DT, but not enzymatically inactive CRM197, induced tumor regression in mice lacking transgenic DTR alleles, indicating that DT's enzymatic activity was required for regression (Fig. 1A). Unlike erlotinib treatment or dox withdrawal, which result in reduced phosphorylated EGFR (pEGFR) levels in tumor-bearing lungs, DT-treated tumors maintained pEGFR levels comparable to those of vehicle- and CRM197-treated tumors (Fig. 1B). Therefore, DT did not directly inhibit EGFR phosphorylation. However, treatment with DT or erlotinib as well as withdrawal of dox reduced the level of pulmonary surfactant-associated protein C (SFTPC, hereafter referred to as SPC), which is expressed by type II lung epithelial cells and tumors derived from them, indicating a reduction of epithelial-derived cells (Fig. 1B) (Politi et al., 2006). We further verified that tumor regression quantified from MRI scans was consistent with Hematoxylin and Eosin (H&E)-stained histological sections of DT-, CRM197-, erlotinib- or vehicle-treated tumors. Although vehicle- and CRM197-treated lung tumor sections were histologically indistinguishable, those treated with DT displayed apoptotic cell fragments and reduced papillary tumor cell clusters (Fig. 1C).

Because tumor-bearing mice lacked a transgenic DTR allele, the cellular target for DT's enzymatic activity was unknown. To test the hypothesis that DT is directly internalized by tumor cells and induces apoptosis, we treated tumor-bearing mice with DT, CRM197, erlotinib or vehicle control for 2 days and quantified apoptotic cells by TUNEL staining (Fig. 1D). We used an EGFR^L858R^-specific antibody to distinguish between tumor (EGFR^L858R^+) and non-tumor (EGFR^L858R^−) cells. Compared to treatment with vehicle control or CRM197, DT (and, to a lesser degree, erlotinib) significantly increased the percentage of apoptotic EGFR^L858R^+ tumor cells (Fig. 1E). Importantly, DT-treated mice had ∼3-fold higher rates of apoptosis in EGFR^L858R^+ tumor cells compared to EGFR^L858R^− cells, whereas CRM197-treated mice displayed no difference (Fig. 1F). These results demonstrate that EGFR^L858R^+ tumor cells are the primary target of DT's enzymatic activity, which results in apoptotic tumor cell death.

### DT-induced tumor regression alters the inflammatory profile of tumor-bearing lungs

**Fig. 2.**
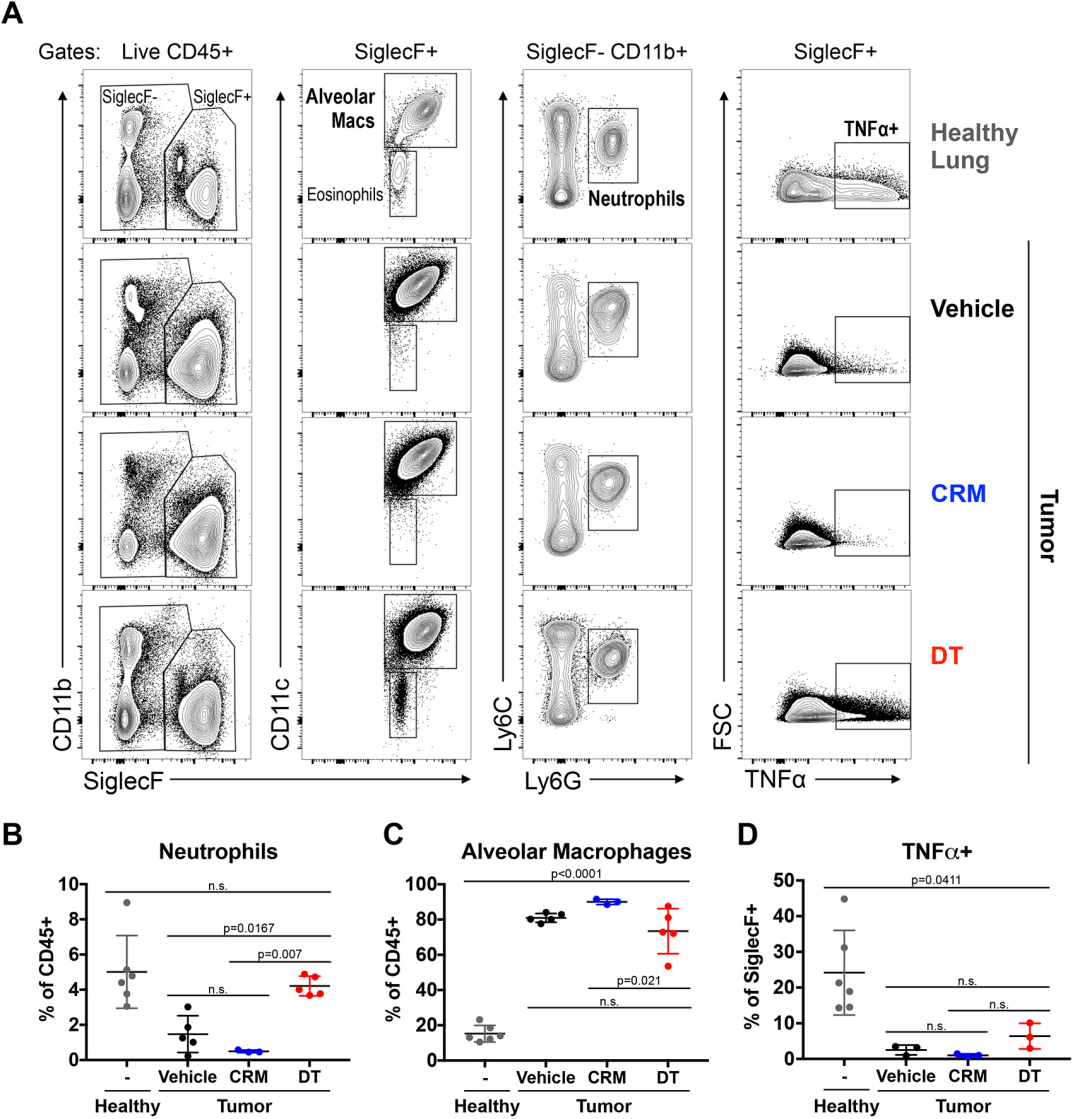
**DT alters the inflammatory profile of tumor-bearing lungs.** Infiltrating immune cells from healthy lungs and from lung tumors derived from FVB *TetO-EGFR^L858R^; CCSP-rtTA* mice treated with PBS (vehicle), CRM197 (CRM) or diphtheria toxin (DT) for 11 days were analyzed by flow cytometry. (A) Representative contour plots demonstrating the gating strategy used to identify distinct myeloid populations and their production of TNFα. (B,C) Percentage of CD11b^+^ Ly6C^+^ Ly6G^+^ neutrophils (B) and CD11b^−^ CD11c^+^ SiglecF^+^ alveolar macrophages (C). (D) Percentage of SiglecF^+^ cells that produce TNFα. Results are shown as mean±s.e.m. with *P*-values calculated using one-way ANOVA (B-D). n.s., not significant.

The inflammatory properties of both DT and CRM197 are well established and form the basis for the use of CRM197 in conjugate vaccines (Schneerson et al., 1980; Shinefield, 2010). To investigate whether inflammation induced by either agent impacts tumor burden, we examined the frequency and function of inflammatory myeloid cells in the tumor microenvironment after treatment with DT, CRM197 or vehicle control. Although vehicle control- and CRM197-treated tumor-bearing lungs had decreased neutrophil frequency compared to healthy controls, DT-induced tumor regression restored neutrophil frequency to the levels in healthy lungs (Fig. 2A,B). Vehicle control- and CRM197- treated tumors had increased alveolar macrophage (AM) frequency compared to healthy controls, yet these AMs produced less TNFα (Fig. 2C,D). However, DT-induced tumor regression failed to restore AM frequency or cytokine production to the levels found in healthy lungs. Thus, DT but not CRM197 altered the inflammatory profile of tumor-bearing lungs, suggesting that these changes are a consequence of tumor regression rather than inflammation induced by the microbial agents.

### Elevated levels of HBEGF confer DT sensitivity to *EGFR*-mutant lung adenocarcinomas

**Fig. 3.**
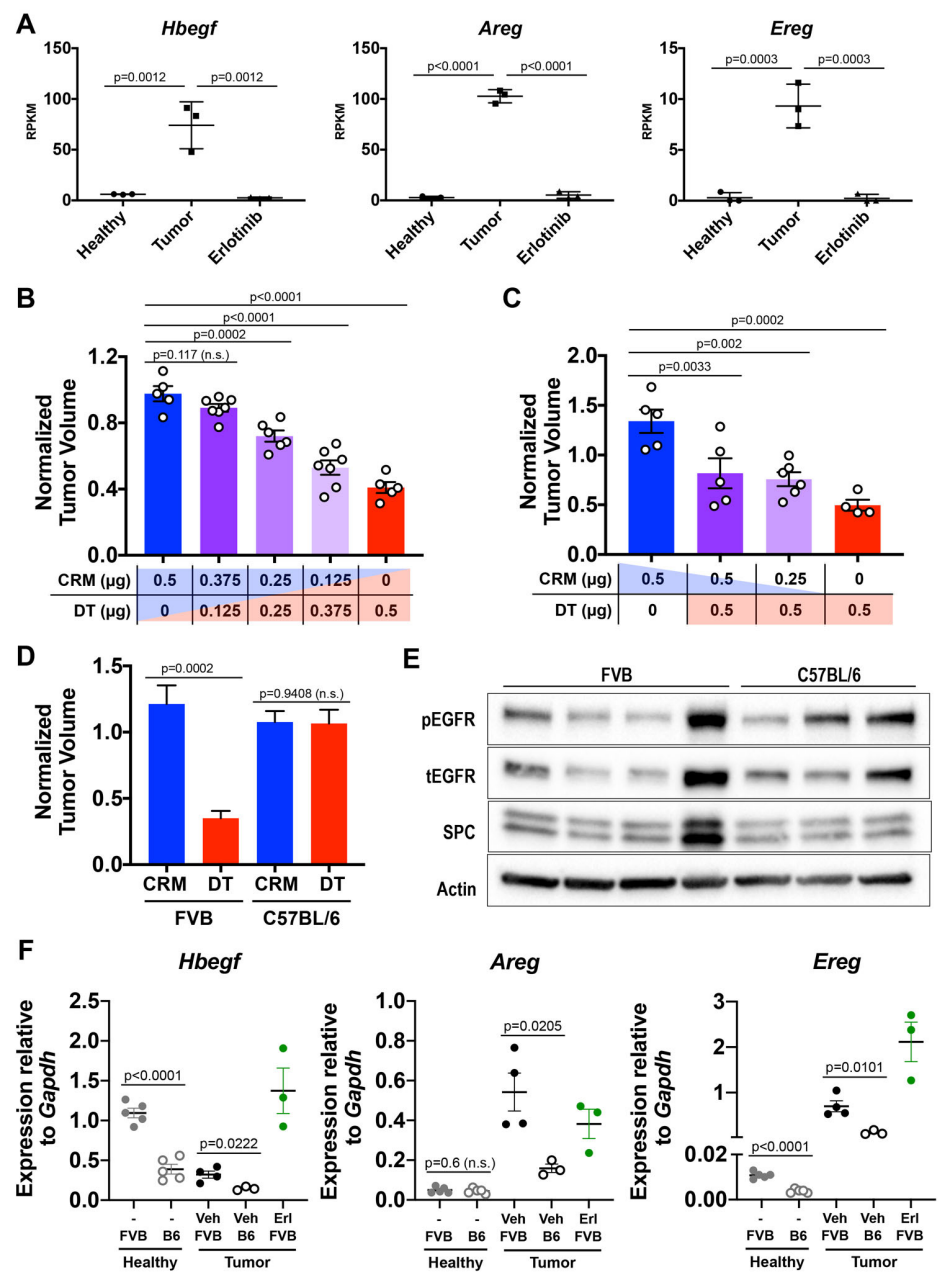
**In FVB mice, HBEGF blockade with the enzymatically inactive DT mutant CRM197 partially abrogates tumor regression mediated by active DT.** (A) mRNA levels expressed in reads per kilobase of transcript, per million mapped reads (RPKM) of *Hbegf*, *Areg*, and *Ereg* in EpCAM+ epithelial cells isolated from lungs of healthy (Healthy), tumor-bearing (Tumor) and erlotinib-treated tumor-bearing (Erlotinib) FVB *TetO-EGFR^L858R^; CCSP-rtTA* mice. (B,C) Normalized tumor volumes quantified from lung MR images of FVB *TetO-EGFR^L858R^; CCSP-rtTA* mice 3 days after treatment with the listed doses of CRM197 (CRM) and/or diphtheria toxin (DT). (D) Normalized tumor volumes quantified from lung MR images of FVB and C57BL/6 *TetO-EGFR^L858R^; CCSP-rtTA* mice 3 days after treatment with DT (FVB *n*=6, C57BL/6 *n*=4), or CRM197 (FVB *n*=3, C57BL/6 *n*=3). (E) Western blot of lung tumor lysates from vehicle control-treated FVB and C57BL/6 *TetO-EGFR^L858R^; CCSP-rtTA* mice immunostained for phosphorylated EGFR (pEGFR), total EGFR (tEGFR) and SPC. Actin was used as a loading control. (F) mRNA expression levels (relative to those of *Gapdh*) of *Hbegf*, *Areg* and *Ereg* in lung tumors of mice as described for E, lung tumors of FVB mice treated with erlotinib (Erl; *n*=3) and healthy lungs (FVB *n*=5, C57BL/6 (B6) *n*=5) from WT mice on doxycycline for 7 days. Results are shown as mean±s.e.m. with *P*-values calculated using unpaired Student's *t*-tests (D) or one-way ANOVA (A-C,F). n.s., not significant.

Next, we investigated the mechanism of DT internalization by *EGFR*-mutant tumor cells *in vivo.* In its membrane-bound state HBEGF is the DT receptor (Naglich et al., 1992); yet, after cleavage from the cell membrane, soluble HBEGF is a high-affinity EGFR ligand that is upregulated by *EGFR*-mutant lung tumors (Jones et al., 1999; Yotsumoto et al., 2017). Oncogenic *EGFR* mutants remain sensitive to ligand binding despite sustaining constitutive ligand-independent phosphorylation (Cho et al., 2013; Red Brewer et al., 2013). Therefore, we hypothesized that *EGFR*-mutant lung tumor cells upregulate murine HBEGF and that this low-affinity DTR could, if expressed at high levels, allow for sufficient DT internalization to induce tumor cell death. Indeed, the entry of one molecule of DT’s catalytic domain was found to be sufficient to kill a cell (Yamaizumi et al., 1978). We found that *Hbegf* as well as two additional EGFR ligands, amphiregulin (*Areg*) and epiregulin (*Ereg*), were specifically upregulated in epithelial cell adhesion molecule (EPCAM)-positive (EpCAM+) lung epithelial cells isolated from tumor-bearing lungs compared to healthy control lungs, and that erlotinib-induced tumor regression restored ligand expression to the levels in healthy lungs (Fig. 3A).

Because enzymatically inactive CRM197 retains HBEGF-binding activity, it can compete with active DT for receptor binding and protect sensitive cells from DT toxicity *in vitro* (Naglich et al., 1992). Therefore, we assessed whether increasing doses of CRM197 could compete with DT for HBEGF binding *in vivo*. First, we treated tumor-bearing mice with a single dose of 0.125-0.5 µg DT combined with CRM197, such that the overall toxin dose was constant across treatment groups, and evaluated tumor burden by MRI on day 3 after treatment (Fig. 3B). This confirmed that DT-induced tumor regression was dose dependent. We then treated tumor-bearing mice with a combination of 0.5 µg DT and increasing (0, 0.25 or 0.5 µg) doses of CRM197. We found that CRM197 partially abrogated DT-mediated tumor regression after 3 days (Fig. 3C). These results suggest that CRM197 competes with DT for HBEGF binding and can protect sensitive tumor cells from DT toxicity.

Although our experiments thus far were performed using FVB mice, many DTR transgenic models are on a C57BL/6 background. Thus, we treated C57BL/6 *TetO-EGFR^L858R^; CCSP-rtTA* tumor-bearing animals with DT and CRM197. Unexpectedly, we found that, in these mice, tumors did not regress (Fig. 3D). Despite similar levels of pEGFR, tumor-bearing whole lungs on a C57BL/6 background displayed reduced expression of *Hbegf*, *Areg* and *Ereg* compared to those on an FVB background (Fig. 3E,F). Healthy lungs from C57BL/6 mice also showed reduced expression of *Hbegf* and *Ereg* compared with expression in lungs from FVB mice (Fig. 3F), indicating that the strain-specific differences in EGFR ligand expression were not tumor specific. In either strain, healthy lungs displayed increased *Hbegf* but decreased *Areg* and *Ereg* expression compared with tumor-bearing lungs; moreover, after treatment with erlotinib, FVB mice showed increased expression of *Hbegf* and *Ereg* (Fig. 3F). These results suggest that, in both healthy lungs and lung tumors, cells other than EpCAM+ epithelial cells (shown in Fig. 3A) also express these ligands. Collectively, these results suggest that, in FVB mice, elevated levels of HBEGF confer sensitivity to DT in *EGFR*-mutant tumors.

## DISCUSSION

Here we show that, in the absence of a transgenic DTR allele, murine lung adenocarcinomas that express human *EGFR^L858R^* are sensitive to DT. We compared the effect of DT with the enzymatically inactive DT mutant CRM197 and found that CRM197 did not induce tumor regression, indicating that enzymatic activity of DT was required for this effect (Fig. 1A). Since both CRM197 and DT can induce inflammation, the requirement for DT enzymatic activity excludes the possibility that inflammation indirectly causes tumor regression. We evaluated whether DT kills tumor cells directly and found the following: (1) EGFR^L858R^+ tumor cells are the primary target for the enzymatic activity of DT, which induces tumor cell apoptosis (Fig. 1D); (2) DT-sensitive *EGFR^L858R^*-induced tumors on an FVB background have higher HBEGF levels compared to DT-insensitive *EGFR^L858R^*-induced tumors on a C57BL/6 background (Fig. 3D); and (3) treatment with CRM197 partially abrogates DT-induced tumor regression (Fig. 3C). Collectively, these results suggest that elevated levels of murine HBEGF confer sensitivity to DT in *EGFR*-mutant tumors.

*In vivo* competition assays helped to exclude potentially confounding factors. Commercial preparations of DT and CRM197 can be contaminated with endotoxin. However, if endotoxin from DT and/or CRM197 does induce tumor regression, increasing doses of either agent should have additive effects, leading to increased regression. DT preparations can also contain nicked DT, in which the catalytic domain has been proteolytically cleaved from the receptor-binding and translocation domains, allowing for indiscriminate entry of the catalytic domain into cells in a receptor-independent fashion. However, receptor-independent entry of nicked DT should not be affected by CRM197. Yet instead of an additive effect (predicted if endotoxin causes regression) or no effect (predicted if nicked DT causes regression), we found that CRM197 partially abrogates DT-induced tumor regression (Fig. 3B,C).

We considered the possibility that the reduced expression of EGFR ligands, including HBEGF, in tumors derived from C67BL/6 mice can be caused by reduced expression levels of the *EGFR^L858R^* transgene in these animals, as strain-specific differences in the epigenetic regulation of transgene expression have been reported (Schumacher et al., 2000). However, we found that, in WT mice lacking the transgene, healthy lung tissue from C57BL/6 mice also showed reduced expression of *Hbegf* compared to healthy lung tissue from FVB mice (Fig. 3F), indicating that the strain-specific difference was not unique to the tumor and not dependent on *EGFR^L858R^* transgene expression. It is possible that epigenetic regulation of EGFR ligands varies across different mouse strains: the accessibility of EGFR ligand (*Hbegf*, *Areg*, *Ereg*) loci could be reduced in C57BL/6 mice, leading to reduced gene expression. Indeed, studies examining DNA methylation patterns across mice of distinct backgrounds have identified strain-specific differences (Zhang et al., 2016).

DT and its derivatives, such as CRM197, have been investigated as cancer therapies. In one early example, 24 of 50 cancer patients experienced reductions in tumor volume upon intravenous DT administration, although the mechanism of response was unknown (Buzzi, 1982). Subsequent efforts have exploited the toxicity of the DT catalytic domain. For example, the DT catalytic and translocation domains were fused to human interleukin-2 (IL2) to form a fusion toxin, denileukin diftitox (Ontak), which selectively kills cells expressing IL2 receptors (Williams et al., 1987; Bacha et al., 1988). Our results suggest that tumors with elevated levels of HBEGF may be uniquely susceptible to DT. In humans, where high-affinity binding between DT and *human* HBEGF can occur, DT delivered in a targeted fashion to tumors expressing HBEGF could be used to provoke rapid tumor cell death while reducing the risks of systemic toxicity, and may warrant further study.

CRM197 has also been investigated as a cancer therapy due to its ability to block HBEGF. In contrast to our findings in the murine setting, human lung cancer xenografts expressing HBEGF were sensitive to CRM197 (Hsieh et al., 2017; Yotsumoto et al., 2017). These results are consistent with the higher affinity of CRM197 for human versus mouse HBEGF. Furthermore, in human lung cancers, HBEGF expression has been positively correlated with levels of EGFR, regardless of *EGFR* mutational status (Hsieh et al., 2017). These results suggest that both *EGFR* WT and mutant lung tumors can produce EGFR ligands, including HBEGF, that promote tumor cell survival via autocrine and/or paracrine EGFR signaling. Therefore, lung cancers with elevated levels of HBEGF may be uniquely susceptible to CRM197 as well as DT.

Transgenic DTR systems for targeted cell ablation *in vivo* have helped to define roles for distinct cell populations in a variety of disease settings. However, our results add to a body of literature demonstrating the biological effects of DT on WT mice, which can be strain specific and must be considered when interpreting results from transgenic DTR models (Meyer Zu Hörste et al., 2010; Goldwich et al., 2012; Chapman and Georas, 2013; Konishi et al., 2017). Importantly, these studies also highlight a unique vulnerability of lung cancers that produce elevated levels of HBEGF.

## MATERIALS AND METHODS

### Animal studies and treatments

Animal studies were performed in compliance with the Institutional Animal Care and Use Committee (protocol 11354; assurance number D16–00416) of Yale University. Animals were housed in a specific pathogen-free facility and fed chow containing 625 ppm doxycycline (dox; Envigo). FVB *TetO-EGFR^L858R^*; *CCSP-rtTA* (Politi et al., 2006) and C57BL/6 *Foxp3^IRES-DTR-GFP^* (Kim et al., 2007) mice have been described. FVB *TetO-EGFR^L858R^*; *CCSP-rtTA* mice were used except where noted in Fig. 3, where we used *TetO-EGFR^L858R^*; *CCSP-rtTA* mice backcrossed to C57BL/6 for ten generations (male and female mice aged 9-16 weeks were used). WT control mice were placed on a dox diet to match tumor-bearing animals. Mice were treated 5 days/week with 25 mg/kg erlotinib (MSKCC Organic Synthesis Core) dissolved in 0.5% methylcellulose via intraperitoneal (i.p.) injection. Mice were treated with 0.5 µg DT or CRM197 (List Biological) via i.p. injection on day 0 only or on days 0, 4, 7 and 10. For *in vivo* competition assays, mice were treated on day 0 with 0.125-0.5 µg DT and/or CRM197 via i.p. injection. MRI scans of mouse lungs were acquired at the Yale Magnetic Resonance Research Center on a mini-4T scanner (Bruker AVANCE) using respiratory gating on mice anesthetized with a steady flow of oxygen and isoflurane. The volume of visible lung opacities (tumors) was calculated using BioImage Suite software (Papademetris et al., 2006). Tumor volumes after treatment with DT, CRM197, vehicle control or erlotinib, or after dox withdrawal, were normalized to the initial tumor volume at the start of treatment.

### Immunohistochemistry and immunofluorescence

Mouse lung tumors were dissected into 4% paraformaldehyde (Electron Microscopy Services), fixed overnight at room temperature and rehydrated in 70% ethanol. Yale Pathology Tissue Services produced paraffin-embedded and H&E-stained sections according to standard methods. After citrate-based antigen retrieval, immunofluorescence staining was performed with anti-EGFR^L858R^ antibody (Cell Signaling #3197) overnight at 4°C and fluorescent secondary for 1 h at room temperature (anti-rabbit AlexaFluor 488, ThermoFisher) with TUNEL staining (ApopTag Red *In Situ* Apoptosis Detection Kit, Millipore #S7165). Total DNA was stained with Hoechst-33342 (ThermoFisher).

### Immunofluorescence quantification

CellProfiler software (Carpenter et al., 2006; Kamentsky et al., 2011) was used to quantify the number and percentage of TUNEL-positive tumor (EGFR^L858R^ positive) or non-tumor (EGFR^L858R^ negative) cells. Between four and ten images each were analyzed from two mice (PBS vehicle control) or three mice (CRM, DT, erlotinib). CellProfiler was programmed to: (1) separate each image into three channels: Hoechst, TUNEL (Rhodamine) and EGFR^L858R^ (AlexaFluor 488); (2) identify primary objects (nuclei) using Hoechst staining and applying global minimum cross entropy thresholding; (3) identify secondary objects (cells) based on EGFR^L858R^-AlexaFluor 488 staining using the global Otsu method with three-class thresholding; (4) identify TUNEL-positive nuclei based on Rhodamine staining using the global Otsu method with three-class thresholding; (5) retrieve the EGFR^L858R^-AlexaFluor 488 integrated intensities of each identified cell; (6) classify and filter cells as either EGFR^L858R^ positive or EGFR^L858R^ negative based on mean EGFR^L858R^-AlexaFluor 488 intensity and; (7) correlate TUNEL-positive nuclei with EGFR^L858R^-AlexaFluor 488-positive and -negative cells.

### Flow cytometry

Mouse lung tumor tissue was minced in RPMI1640 with 2% FBS, 0.5 mg/ml collagenase IV (Sigma), and 1 µg/ml DNase (Sigma) and digested at 37°C for 45 min. Samples were filtered through a 70 µm cell strainer, incubated with ammonium-chloride-potassium lysis buffer (ThermoFisher) for 3 min, washed with PBS, and resuspended in RPMI1640 with 2% FBS. For intracellular cytokine staining, single-cell suspensions were incubated in RPMI1640 with 10% FBS and brefeldin A (ThermoFisher) at 37°C for 5 h. Single-cell suspensions were washed with PBS with 2% FBS, incubated with α-FcɣRIII/II antibody (2.4G2) on ice for 15 min, then stained on ice for 30 min. For intracellular staining, samples were fixed and permeabilized using the eBioscience Foxp3/Transcription Factor Staining Buffer Set (ThermoFisher) and then stained on ice for 45 min. The following antibodies were used for surface staining: CD45 BV711 (1:400; #103147), CD3 APC-Cy7 (1:100; #100222), CD4 PerCP (1:200; #100538), CD8a BV605 (1:200; #100744), CD11b APC-Cy7 (1:200; #101226), CD11c PE-Cy7 (1:200; #117310), Ly6G PerCP (1:200; #127653), Ly6C BV605 (1:200; #128036) (all from BioLegend) and SiglecF BV480 (1:400; BD Biosciences #746668). The following antibodies were used for intracellular staining: Foxp3 AF488 (1:100; #126406) and TNFα PE (1:300; #506306) (both from BioLegend). Dead cells were excluded using the LIVE/DEAD Fixable Red Dead Cell Stain Kit (ThermoFisher). Flow cytometry data were acquired on an LSR II (BD) with FACSDiva and analyzed with FlowJo (TreeStar).

### Immunoblotting

Mouse lung tumors were flash frozen in liquid nitrogen, crushed and lysed in RIPA buffer containing protease inhibitor. Equal amounts of total protein were separated by gel electrophoresis on Mini-PROTEAN GTX 4-20% gels (Bio-Rad), transferred onto nitrocellulose (Bio-Rad) and probed with the following antibodies: phospho-EGFR (Tyr1068) (Cell Signaling #3777), EGFR (Cell Signaling #2232), SPC (Abcam #90716), and β-actin (Santa Cruz #47778).

### RNA extraction and real-time quantitative PCR

Mouse lung tumors were flash frozen in liquid nitrogen and crushed. RNA was extracted using the RNeasy Mini kit (QIAGEN). cDNA was synthesized using the SuperScript III First-Strand Synthesis System (ThermoFisher) and quantitative RT-PCR was performed on a ViiA 7 Real-Time PCR System (Applied Biosystems) using Power SYBR Green PCR Master Mix (Applied Biosystems). Transcript levels were normalized to those of *Gapdh.* The following primers were used: *Hbegf* (forward, 5′-CGCGTTGGTGACCGGTGAGAG-3′; reverse, 5′-CCCGTGGGTAGCAGCTGGTTT-3′); *Areg* (forward, 5′-GCTGAGGACAATGCAGGGTA A-3′; reverse, 5′-GTGACAACTGGGCATCTGGA-3′); *Ereg* (forward, 5′-TGCTTTGTCTAGGTTCCCACC-3′; reverse, 5′-GGCGGTACAGTTATCCTCGG-3′); *Gapdh* (forward, 5′-ATGGTGAAGGTCGGTGTGAA-3′; reverse, 5′-TGGAAGATGGTGATGGGCTT-3′) (Kefaloyianni et al., 2016).

### RNA sequencing

RNA sequencing performed on lung epithelial cells isolated from healthy, tumor-bearing and erlotinib-treated tumor-bearing mice as described by Ayeni et al. (2019) was analyzed. Briefly, EpCAM^+^ CD45^−^CD11c^−^ lung epithelial cells were sorted on a FACS Aria (BD) at the Yale Flow Cytometry Core. RNA was extracted and purified using the Arcturus PicoPure RNA isolation kit. RNA sequencing was performed on the Illumina HiSeq 2000 platform at the Yale Stem Cell Center Genomics and Bioinformatics Core. Reads were aligned using bowtie2 (Langmead and Salzberg, 2012) to the GRCm38 (mm10) reference genome. Normalized reads are shown in reads per kilobase of transcript, per million mapped reads (RPKM).

### Statistical analysis

Analyses were performed using GraphPad Prism software (v7.0a); results are shown as mean±s.e.m. with *P*-values from unpaired Student's *t*-tests or one-way ANOVA, with *P*<0.05 considered significant.

## Supplementary Material

Supplementary information
