# Supplementary material for: Elevated murine HB-EGF confers sensitivity to diphtheria toxin in EGFR-mutant lung adenocarcinoma
Source: Dis Model Mech. 2021 Nov 15;14(11):dmm049072. doi: 10.1242/dmm.049072 (PMC8617309; doi:10.1242/dmm.049072)
Supplement: Supplementary information [file dmm-14-049072-s1.pdf]

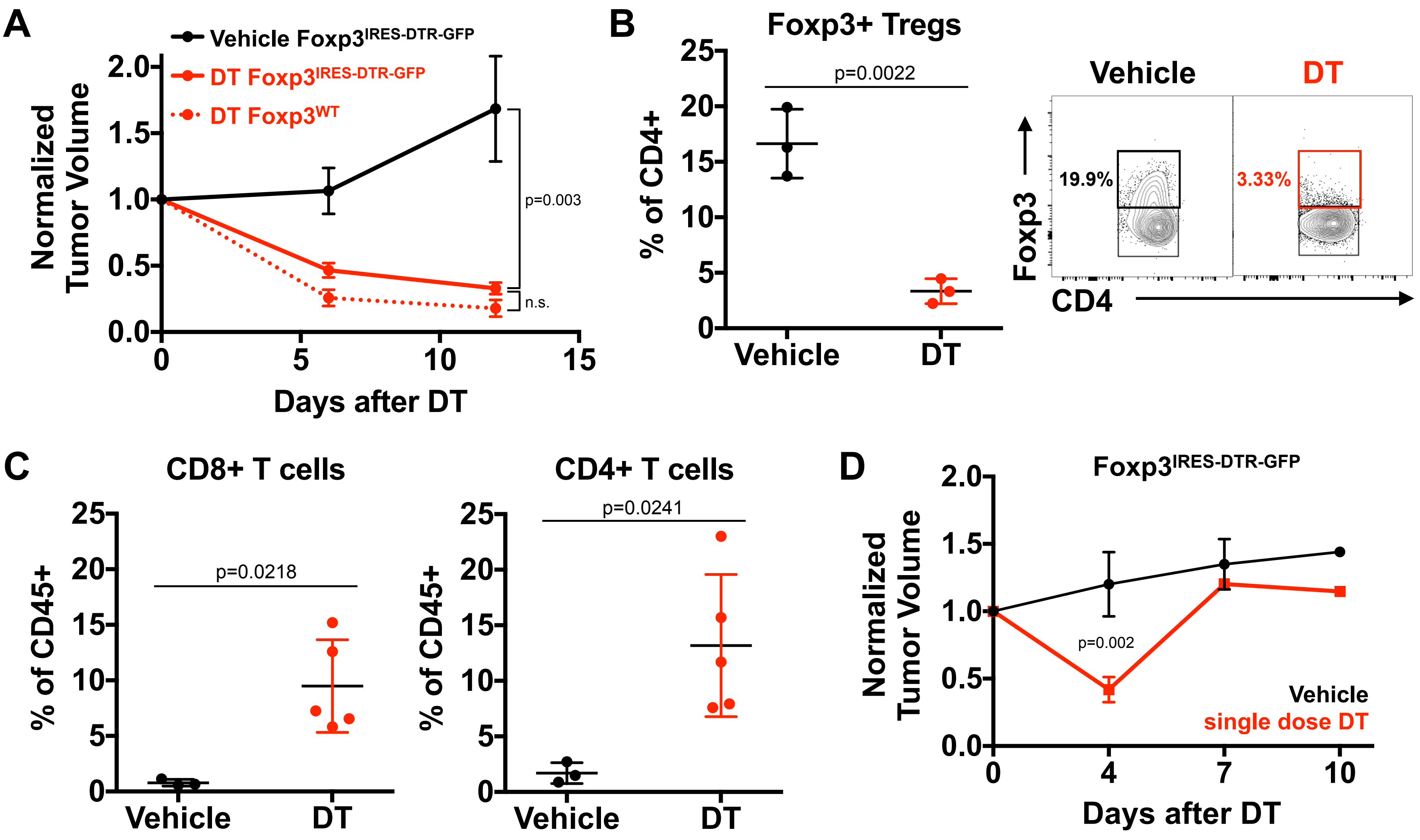

Fig. S1. DT treatment in Foxp3^{DTR} tumor-bearing mice induces T$_{reg}$ depletion and transient tumor regression. (A) Normalized tumor growth curves quantified from lung MR images of *CCSP-rtTA; TetO-EGFR^{L858R}; Foxp3$^{IRES-DTR-GFP}$* mice treated with vehicle (n=7) or DT (n=10) and *CCSP-rtTA; TetO-EGFR^{L858R}; Foxp3^{WT}* mice treated with DT (n=2) for 12 days. **(B, C)** Lung tumor infiltrating immune cells were analyzed by flow cytometry after 12 days of DT or vehicle treatment. Quantification of the frequency of **(B)** Foxp3^{+} Tregs (with representative contour plots) and **(C)** CD8^{+} and CD4^{+} T cells. **(D)** Normalized tumor growth curves quantified from lung MR images of *CCSP-rtTA; TetO-EGFR^{L858R}; Foxp3$^{IRES-DTR-GFP}$* mice treated with a single dose of vehicle (n=2) or DT (n=4) on day 0.

Disease Models & Mechanisms • Supplementary information
